# Hydrogels, Oleogels and Bigels as Edible Coatings of Sardine Fillets and Delivery Systems of Rosemary Extract

**DOI:** 10.3390/gels8100660

**Published:** 2022-10-16

**Authors:** Aikaterini Kanelaki, Konstantina Zampouni, Ioannis Mourtzinos, Eugenios Katsanidis

**Affiliations:** Department of Food Science and Technology, School of Agriculture, Faculty of Agriculture, Forestry and Natural Environment, Aristotle University of Thessaloniki, 54124 Thessaloniki, Greece

**Keywords:** sardine fillets, edible coatings, plant extracts, hydrogel, oleogel, bigel, lipid oxidation

## Abstract

Edible coatings provide an alternative way to reduce packaging requirements and extend the shelf life of foods by delaying oxidation and microbial spoilage. Hydrogels, oleogels and bigels were applied as coatings on fresh sardine fillets. The effectiveness of these coatings as delivery systems of rosemary extract (RE) was also evaluated. Three groups of sardine fillet treatments were prepared: (i) the control (C), which comprised sardine fillets without coating, (ii) sardine fillets with plain hydrogel (H), oleogel (O) or bigel (BG) coatings, and (iii) sardine fillets with RE incorporated into the H, O and BG coatings. The different treatments were evaluated for lipid oxidation (TBA test), total volatile basic nitrogen (TVB-N) and microbiological growth during cold storage at 4 °C. Results showed that hydrogel, oleogel and bigel coatings delayed oxidation. The incorporation of RE into coatings significantly retarded lipid oxidation but did not affect the proliferation of microorganisms during storage. When RE was incorporated in the oleogel phase of the bigel coating, it produced significantly lower TVB-N values compared to the control and BG treatments. The incorporation of RE into the oleogel phase of the bigel coating may be a promising method of maintaining the storage quality of the sardine fillets stored at refrigerated temperatures.

## 1. Introduction

*Sardina pilchardus*, commonly known as European pilchard, is one of the most commercially exploited fish species, with significant nutritional and economic importance. It is rich in polyunsaturated fatty acids, mainly omega-3 fatty acids, and comprises an excellent source of high biological value proteins, minerals and vitamins for human consumption [1]. However, owing to the great amount of omega-3 and omega-6 fatty acids, sardines are highly susceptible to oxidation [2], resulting in degradation of organoleptic characteristics, loss of nutritional value and shortening of shelf life. During storage, fresh sardines are particularly vulnerable to deterioration due to the combined effect of the metabolic activity of microorganisms and the enzymatic processes [3]. Therefore, degradation of sardine quality occurs rapidly throughout handling and storage periods, leading to limited shelf life.

Edible coatings present an effective and environmentally friendly alternative to enhance quality and extend food preservation during refrigerated storage. Coatings can be prepared from various compounds, such as carbohydrates (starch, cellulose, alginates), proteins (gelatin, whey protein, casein, zein) and lipids (waxes, oils, fats) [4]. Therefore, systems such as hydrogels, oleogels or the combination of them, as bigels, could be used as edible coatings. The process of coating includes the direct immersion of the food in a liquid solution [5]. Edible coatings can act as a barrier to the ingress of oxygen and water in food, resulting in slowing oxidation reactions and retaining moisture [6]. Various edible coatings have been studied for the preservation of fishery products during refrigerated storage, such as chitosan coatings on Indian oil sardine (*Sardinella longiceps*) [7], chitosan-gelatin coatings on shrimp (*Litopenaeus vannamei*) [8], and sodium alginate or whey protein coatings on rainbow trout (*Oncorhynchus mykiss*) fillets [9,10]. The formation of a barrier between atmospheric oxygen and food products can retard the oxidation process and extend the shelf-life of foods. Thereby, the application of an edible coating could be effective, especially during the storage of fishery products, as it could delay microbial growth and oxidative deterioration [11]. The application of whey protein-based coatings, for example, has been reported to inhibit lipid oxidation in Atlantic salmon (*Salmo salar*) fillets [12]. Regarding microbial stability, the spoilage of fishery products mainly takes place due to the growth of Gram-negative, psychotropic bacteria [13]. *Pseudomonas* spp. is considered the most important psychotropic microorganism, causing fish spoilage when stored under aerobic low temperatures [14].

Hydrogels are three-dimensional, hydrophilic macromolecular networks formed by interactions among the polymeric chains of a gelling agent, retaining large amounts of water [15]. In addition, most hydrogels are characterized as reversible, with the capability to alter their rheological properties due to changes in external conditions (temperature, pH, ionic solution strength, etc.) [16]. Gelatin is an ideal coating material due to its gelling ability and resistance to dehydration, light and oxygen [17].

Oleogels are three-dimensional, anhydrous, viscoelastic gels developed through the addition of low molecular weight or polymeric structures in edible oils, leading to the structuring of the continuous phase of the system [18]. Waxes, fatty acids and alcohols, lecithin, monoglycerides (MGs) and a mixture of phytosterols with oryzanol [19] or MGs [20] have been used as low molecular weight oleogelators [21]. Studies have demonstrated that structured oil could efficiently replace animal fat in foods [22,23,24,25,26]. Oleogel and oleogel-based systems have great potential as delivery vehicles of lipophilic bioactive compounds [21,27].

Bigels (hybrid gels) are biphasic systems where both the lipid and the aqueous phase are structured in the form of oleogel and hydrogel, respectively [28]. Technically, bigels resemble emulsions that include a gel network in both their aqueous and lipid phases, but they confer better physicochemical stability over time compared to plain emulsions [29]. Bigels are structured through the dispersion of one phase into the other, mostly forming oleogel-in-hydrogel bigel systems [30]. The fact that bigels consist of two structured phases provides the advantage of the controlled delivery of both hydrophilic and lipophilic bioactive substances [31]. In addition, their relatively easy preparation methods [32], spreadability [31], extended shelf-life, and the stability for 6–12 months at room temperature [33] give these systems the opportunity to be utilized as edible coatings for foods. Currently, some food-grade bigels have been used as potential fat substitutes in food products [34,35].

Increasing consumer demands for safer, high-quality food products with prolonged shelf lives have led the food industry to the broad use of chemical preservatives, ensuring the microbiological and oxidative stability of perishable foods. However, the use of synthetic preservatives has raised concerns regarding potential health risks [36,37,38]. A new trend in the food industry, called green consumerism, aims to develop alternative methods of food preservation and is more focused on using natural ingredients [39]. Specifically, essential oils and plant extracts attract interest as prospective preservatives due to their low toxicity, high bioaccessibility and wide acceptance by consumers [40]. The functionality of natural extracts and essential oils relies on inhibiting the growth of microorganisms (food safety) and controlling the natural spoilage processes (food preservation) [41]. In general terms, incorporating plant extracts into edible coatings could delay or prevent food deterioration, by controlling lipid oxidation or microbial growth. Thus, edible coatings enriched with plant extracts could be an approach to enhance the quality and extend the shelf life of perishable foods, such as sardine fillets.

Rosemary (*Rosmarinus officinalis*, L.) is a common aromatic herb, approved as a natural food antioxidant in the EU primarily due to its high concentration of antioxidant compounds, such as rosmarinic acid, carnosol and carnosic acid [42]. Rosmarinic acid is a more hydrophilic substance compared to carnosol and carnosic acid, which are more soluble in hydrophobic solvents [43]. The antioxidant activity is achieved by donating hydrogen atoms or electrons, which scavenge the free radicles. The rosmarinic acid exhibits strong antioxidant activity due to its structure, which is comprised of two phenolic rings [44]. In addition, the carnosic acid and carnosol, typically found in rosemary extracts, protect against oxidation progress by stabilizing the hydroperoxides [45]. Specifically, these phenolic compounds inhibit the decomposition of hydroperoxides into active forms, such as malonaldehyde, and create a complex with Fe^2+^, ensuring the prevention of hydroxyl radical formation [46]. Sarabi et al. (2017) reported the antioxidant effect of RE on coated fried Escolar (*Lipidocybium flavobrumium*) fish fillets during frozen storage [47]. Peiretti et al. (2012) investigated the effects of rosemary oil (RO) on the oxidative stability of minced rainbow trout at 4 °C and found that treatments enriched with RO had lower TBARS values than the control [48]. Furthermore, ice containing RE improves the oxidative stability and extension of the shelf life of sardine (*Sardinella aurita*) [49]. Moreover, various microorganisms are also vulnerable to the activity of rosemary oil, as it contributes to the increased permeability of the microbial cell membrane [50]. According to Klančnik et al. (2009), the antimicrobial activity could be affected by the concentration and the chemical nature of the phenolic compounds in RE [51]. The antimicrobial activity of extracts is mainly attributed to phenolic compounds, which can disrupt the bacteria’s cell wall and penetrate the cell, leading to protein denaturation, cell membrane destruction and cell death. Considering the above, the antimicrobial activity of extracts is expected to be lower against Gram-negative bacteria because the additional outer membrane of Gram-negative bacteria surrounds their cell wall, restricting the diffusion of hydrophobic compounds through the membrane and reducing the effect of the antimicrobial compounds [52]. The direct application of rosemary extract in fish flesh was effective in delaying lipid oxidation of gilt-head sea bream (*Sparus aurata*) and salmon (*Salmo salar*) fillets [53,54].

To the best of our knowledge, the application of gelatin hydrogels, sunflower oil oleogels, and bigels with or without rosemary extract for the preservation of the quality of sardine fillets has not been studied to date. Thus, the objective of the present study was to evaluate the efficacy of hydrogels, oleogels, and bigels as edible coatings and potential delivery systems of rosemary extract by examining the chemical and microbiological attributes of coated sardine fillets during refrigerated storage.

## 2. Results and Discussion

### 2.1. Evaluation of Gels as Edible Coatings

#### 2.1.1. Thiobarbituric Acid (TBA) Analysis

**Figure 1:**
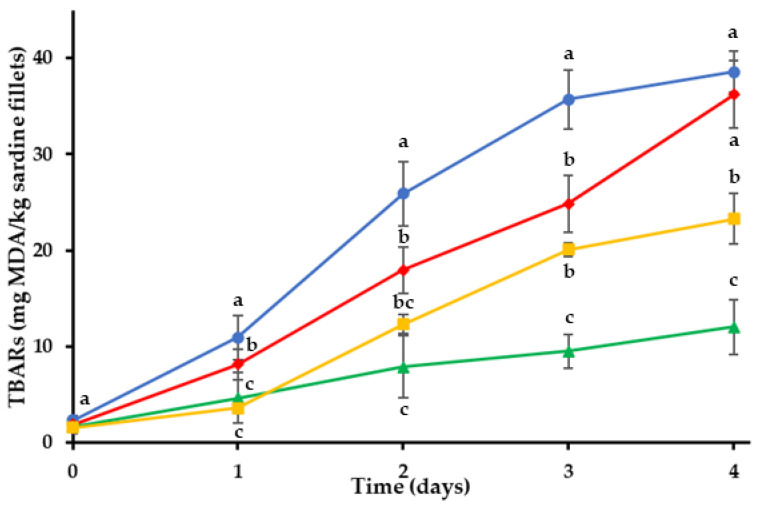
Oxidation values of sardine fillets with edible coatings during storage at 4 °C. (●) C: control-uncoated fillets; (♦) H: hydrogel coating; (▲) O: oleogel coating; (■) BG: bigel coating. ^a–c^ Different letters in the same day indicate significant differences (*p* < 0.05).

Changes in TBARs of the sardine fillets throughout the storage period are shown in Figure 1. Initial TBARs were found to be 1.55–2.35 mg MDA/kg. Control treatment (C) had the highest TBARs during storage compared to coated treatments (*p* < 0.05). TBARs of C increased faster compared to H, O and BG treatments. The oxidation process followed an increasing course in all the sardine treatments up to 4th day, but lower values were observed for the coated fillets (H, O and BG). The application of the different edible coatings on sardine fillets showed statistically significant inhibition of lipid oxidation (*p* < 0.05). Treatment O exhibited the lowest TBARs during the storage time, which were recorded as 12.01 ± 2.88 mg MDA/kg on 4th day. The data illustrated in Figure 1 indicate that the oleogel coating was effective in retarding the production of secondary lipid oxidation products in sardine fillets by acting as a barrier to oxygen permeation and slowing oxygen diffusion into the fish. In addition, the bigel (containing 80% hydrogel and 20% oleogel phase) was a much more effective oxygen barrier that the plain hydrogel coating (H), which exhibited the least effectiveness against lipid oxidation.

Thiobarbituric acid values (TBARs) provide a measure of the concentration of secondary lipid oxidation products due to the auto-oxidation of peroxides to aldehydes and ketones [55]. Mendes et al. (2008) reported that the partial dehydration process of the fish and the oxidation of unsaturated fatty acids contributed to the increase in TBARs under chilled storage [56]. The edible coatings, in addition to providing a barrier to oxygen permeation, also prevented dehydration of the fillet surface, thus protecting the sardine fillets from oxidative deterioration.

#### 2.1.2. Microbiological Analysis

The changes in psychotropic counts (PTC), *Enterobacteriaceae* and *Pseudomonas* spp. of sardine fillets during the refrigerated storage are shown in Table 1. The initial low microbial counts indicated that the fish were of good microbiological quality. The PTC of all the examined treatments increased gradually, as the storage temperature was optimal for these bacteria to proliferate [57]. The hydrogel coating (H) resulted in lower (*p* < 0.05) microbial counts than C and other coated treatments (O and BG) throughout the storage period (Table 1). Control and coated treatments reached 8–9 log (CFU/g) in PTC on the seventh day of storage.

During the storage period, the counts of *Pseudomonas* spp. showed an increasing trend for C and coated treatments (H, O and BG) (Table 1). The increase was significantly lower (*p* < 0.05) for H treatments compared to C. The observed antimicrobial activity of gelatin hydrogel could be related to the oligopeptide chains derived from the hydrolysis of collagen for the formation of gelatin and the presence of side-chain amino groups [58]. Analogous antimicrobial properties have also been reported for other hydrolyzed muscle proteins [59]. The population of *Pseudomonas* spp. of the C, H, O and BG treatments reached 9.83, 9.25, 9.40 and 9.74 log (CFU/g), on 7th day, respectively.

*Enterobacteriaceae* bacteria constitute an indicator of the deterioration of the hygienic conditions of fish. The application of coatings affected *Enterobacteriaceae’s* growth (*p* < 0.05). Initial *Enterobacteriaceae* counts were about 1.9–2.5 log (CFU/g). After seven days of refrigerated storage, *Enterobacteriaceae* reached approximately 8.5 log (CFU/g) for uncoated and BG sardine fillets. Generally, the hydrogel and oleogel coatings showed some antimicrobial activity against this microorganism. The H treatment exhibited the lowest *Enterobacteriaceae* counts up to the fifth day of refrigerated storage, in agreement with the previous observations for PTC and *Pseudomonas* spp.

### 2.2. Evaluation of Gels as Delivery Systems of Rosemary Extract

#### 2.2.1. Thiobarbituric Acid (TBA) Analysis

**Figure 2:**
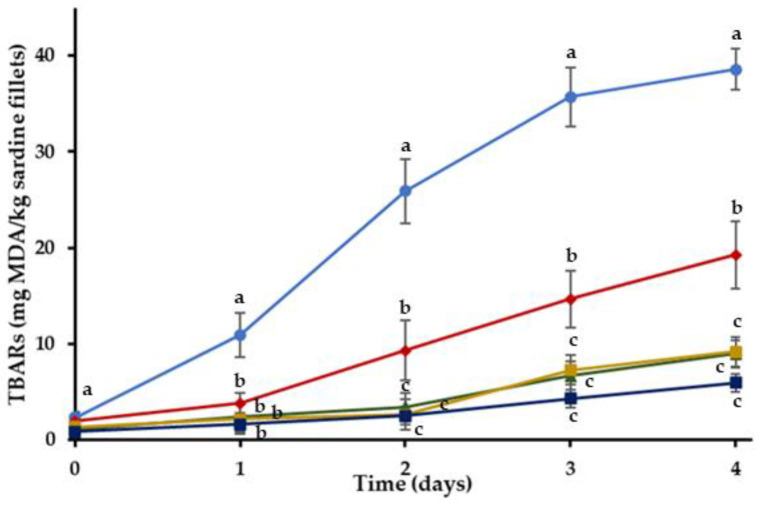
Oxidation values of sardine fillets with incorporated RE coating during storage at 4 °C. (●) C: control-uncoated fillets; (♦) HR: hydrogel coating with RE; (▲) OR: oleogel coating with RE; (■) BGHR: bigel coating with added RE in the aqueous phase; (■) BGOR: bigel coating with added RE in the lipid phase. ^a–c^ Different letters in the same day indicate significant differences (*p* < 0.05).

TBARs of C and treatments with RE during storage at 4 °C are shown in Figure 2. MDA measurements showed that the initial oxidation of the sardine fillets was low on 0 day. It was found that incorporating RE in edible coatings affected TBARs development (*p* < 0.05). There was a progressive increase of lipid oxidation in C and the treatments with the RE-enriched coatings throughout storage. However, significantly lower (*p* < 0.05) TBARs were found for HR, OR, BGHR and BGOR treatments in comparison with the C treatments. Moreover, lower oxidation levels were measured in coated treatments enriched with RE (HR, OR, BGHR and BGOR) compared to the coated treatments without RE (H, O, BG). Results support that incorporating RE in different types of coatings can retard the oxidative deterioration of the refrigerated sardine fillets.

It has been established by several researchers that the incorporation of phenolic compounds into protein-based coatings may lead to the formation of hydrogen bonds between phenols and protein functional groups, resulting in the improvement of the mechanical attributes and water barrier properties of these type of coatings [60]. Thereby, the incorporation of the phenolic-rich RE in HR, is likely to enhance the barrier properties of the gelatin-based coating, delaying the oxidation deterioration of the fillets.

**Figure 3:**
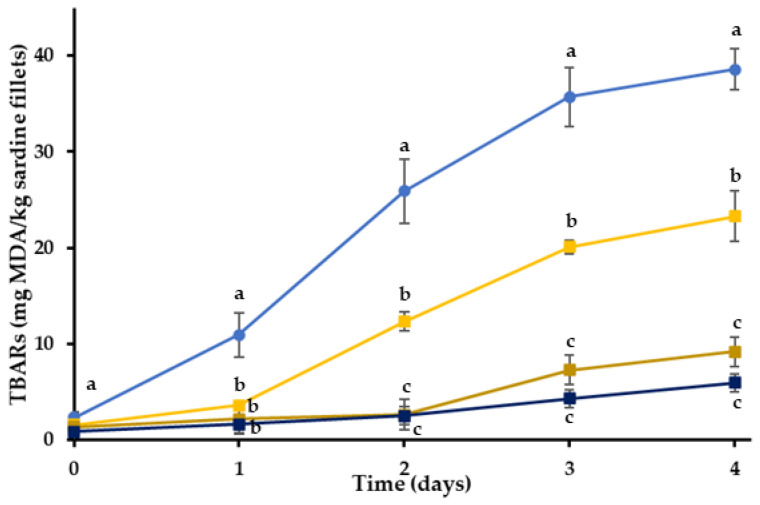
Oxidation values of sardine fillets with or without incorporated RE coating during storage at 4 °C. (●) C: control-uncoated fillets; (■) BG: bigel coating; (■) BGHR: bigel coating with added RE in the aqueous phase; (■) BGOR: bigel coating with added RE in the lipid phase. ^a–c^ Different letters in the same day indicate significant differences (*p* < 0.05).

For the evaluation of the functionality of RE in the different phases (aqueous or lipid), the extract was incorporated into either the hydrogel or the oleogel phase of the bigel coatings. The TBARs of the sardine fillets with bigel coatings were significantly lower than the control samples (*p* < 0.05) and the RE showed a strong antioxidant activity (Figure 3). Even though the oxidation levels of the BGOR treatment were lower in absolute values than the BGHR treatment after the second day of storage, these differences were not statistically significant (*p* > 0.05). Therefore, the incorporation of RE into the oleogel or the bigel inhibited the lipid oxidation of sardine fillets in a comparable manner. The main active ingredient of the RE is rosmarinic acid, a water-soluble compound that would tend to partition into the aqueous phase of the bigel, even when the RE was incorporated into the lipid phase. Apart from rosmarinic acid, rosemary extracts also contain less polar ingredients, like carnosol (a phenolic diterpene) [61], that can be found in a propylene glycol extract [62]. When the RE is added in a complex matrix such as a bigel, the less polar ingredients could be transferred to the lipid fraction and a part of rosmarinic acid could partition into the aqueous phase of the bigel during mixing. It should be noted that the two phases of the bigel are mixed together when they are in a molten state, facilitating the partitioning process. The antioxidant activity of RE probably depends on the polarity of the edible coatings, as the coatings with a lipid phase seemed to be more efficient as delivery and controlled release systems of the RE. The better performance of the OR, BGHR and BGOR treatments compared to the HR could also be attributed to the better oxygen barrier properties of these gels compared to the gelatin hydrogel (H), as discussed in the previous section. Furthermore, these results could be associated with the fact that the diffusion rate of RE is slower in the oleogel system resulting in a gradual release of the antioxidant compound throughout the whole experiment [63].

#### 2.2.2. Microbiological Analysis

As previously observed in the other treatments, the psychotropic counts (PTC), *Pseudomonas* spp. and *Enterobacteriaceae* of sardine fillets increased progressively with the storage time for HR, OR, BGHR and BGOR. The initial PTC of sardine fillets was 2.90–3.77 log (CFU/g) on day 0. On day 7, the PTC of C, HR, OR, BGHR and BGOR treatment reached 9.86, 8.00, 9.00, 9.42 and 9.15 log (CFU/g), respectively (Table 1). Lower final PTC of sardine fillets were observed for HR treatment compared to C and other coated treatments, while in the early days of storage, lower values were observed in bigel-coated treatments. Refrigerated storage resulted in an increase in *Pseudomonas* spp. and *Enterobacteriaceae* of the fillets with RE coating (Table 1). Even though the RE treatments had lower microbial counts than the plain coatings, these differences were not statistically significant (*p* > 0.05). It has been reported that the incorporation of 1.5% rosemary extract in refrigerated Nile tilapia (*Oreochromis niloticus*) fillets had no protective effect against *Pseudomonas* spp. [39].

#### 2.2.3. Total Volatile Basic Nitrogen (TVB-N)

**Figure 4:**
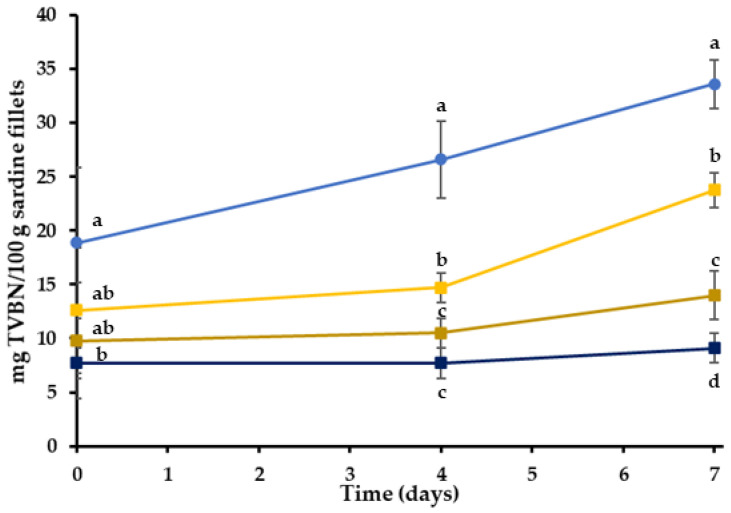
Total volatile basic nitrogen (TVBN) in the treatment during chilled storage (4 °C). Results have been expressed as mg of TVBN per 100 g of sardine fillets. (●) C: control-uncoated fillets; (■) BG: bigel coating; (■) BGHR: bigel coating with added RE in the aqueous phase; (■) BGOR: bigel coating with added RE in the lipid phase. ^a–d^ Different letters in the same day indicate significant differences (*p* < 0.05).

Small-sized molecules, such as volatile nitrogenous compounds, biogenic amines and organic acids, are produced by the metabolism of basic spoilage microorganisms in fresh fish and serve as spoilage indicators. Specifically, total volatile basic nitrogen (TVB-N) represents many different nitrogenous compounds, such as ammonia and primary, secondary and tertiary amines, formed by enzymatic action, and is widely used as an important indicator of fish and seafood deterioration [64,65]. According to Connel (1995), the concentration of TVB-N in a fresh fish is typically between 5–20 mg TVB-N/100 g, while the acceptability limit is 30–35 mg TVB-N/100 g of fish flesh [66]. The TVB-N determination revealed significant differences between the control and bigel treatments. TVB-N concentrations of the various treatments are shown in Figure 4.

On the fourth day of storage, the TVBN values of C, BG, BGHR and BGOR were 26.6, 14.7, 10.5 and 7.7 mg/100 g, respectively. The bigel coatings significantly delayed TVB-N formation compared to the control (C) (Figure 4) (*p* < 0.05). According to the results, the TVB-N content of all treatments gradually increased during storage, but the level of 30 mg/100 g was exceeded only in treatment C, at the end of the storage period. The BGHR and BGOR treatments reached significantly lower TVB-N values of 14.0 mg/100 g and 9.1 mg/100 g in comparison to C treatments (*p* < 0.05). Based on the TVB-N results, it can be concluded that the RE has a more intense effect on inhibiting the TVN-N production when added in the oleogel phase of the bigel (BGOR). Similar studies reported that fish fillets coated with edible films containing extracts or essential oils in multiple concentrations showed lower TVB-N values than non-coated samples stored under refrigeration [67]. Based on these observations, it can be concluded that, even if the composition of BGHR and BGOR is identical, the incorporation phase of RE plays an essential role in the functionality of the edible coating.

## 3. Conclusions

Hydrogels, oleogels, and bigels were applied as edible coatings of sardine fillets. The edible coatings had a significant effect on inhibiting sardine fillets oxidation, while they offered a marginal benefit in microbial growth control. These gel systems were also evaluated for their functionality as delivery systems of rosemary extract. Sardine fillet spoilage, as indicated by lipid oxidation and TVB-N levels, was further limited when rosemary extract was added into the edible coatings. Bigels offered good functionality as delivery systems of rosemary extract. Delivery system functionality can be differentiated, depending on the polarity of the bioactive compounds and whether the bioactive compounds are solubilized in the aqueous or the lipid phase of the bigels. The efficiency of RE increased when it was incorporated in the oleogel phase of bigel, inhibiting the oxidative changes and the production of TVB-N of the coated fillets. Gels used as edible coatings could extend the shelf life of fishery products, regarding the lipid oxidation process. Bigels in particular can be used as coatings and potential delivery systems of bioactive substances in sardine fillets during cold storage.

## 4. Materials and Methods

### 4.1. Sardine Fillet Preparation

Sardines were purchased fresh from the local fish market (Nea Mihaniona, Greece) and transferred to the laboratory in a cooled box covered with crushed ice within 30 min. Upon arrival, each fish was eviscerated, filleted by hand, and carefully washed with cold water. Two fillets were obtained from each fish after removing the head and bones. The weight of each sardine fillet was approximately 8 g.

### 4.2. Preparation of Coating Solution and Treatment of Fish Fillets

To prepare the gelatin hydrogel, 10% *w*/*w* gelatin from bovine and porcine bones (Type A Gelatin, Sigma-Aldrich, Germany) was hydrated under constant stirring in distilled water at room temperature for 10 min. Then, the gelatin suspension was heated at 80 °C for 10 min until gelatin was fully dissolved.

For oleogel preparation, sunflower oil (Minerva S.A., Metamorphosis, Greece) was heated at 90–95 °C, and then 15% *w*/*w* monoglycerides (MGs, HARI 95 distilled monoglycerides Rikevita SDN BHD-Malaysia) were added to the hot sunflower oil as structurants. The mixture was continuously stirred at 90–95 °C for 60 min [23].

Bigels were prepared by slowly incorporating the molten sunflower oil oleogel into the gelatin hydrogel at 70 °C at a 20:80 ratio, under constant stirring for 15 min, using a magnetic stirrer at 300 rpm. The concentrations and the mixing ratio of hydrogels and oleogels were selected so that the coatings remained fluid at a temperature (45 °C) that did not affect the viability of the natural microflora of the sardine fillets.

A commercially produced rosemary extract (RE) (AquaROX, Vitiva, Slovenia) was incorporated at a concentration of 2% into the individual gels (at 50 °C) under constant stirring. The commercial rosemary extract (RE) solution consisted of 90% propylene glycol and 10% rosemary extract, with rosmarinic acid as the main active ingredient, according to manufacturer’s specifications. The same concentration of propylene glycol (2%) was added to all other coatings (gels) that did not contain the extract, to ensure the greatest possible uniformity among coatings.

Sardine fillets were randomly separated into three groups. The first group of fillets was untreated and uncoated, and was used as the control treatment (C). A part of the second group of fillets was coated by dipping in gelatin hydrogel (H), another in sunflower oil oleogel (O) and another in bigel (BG). The direct coating was applied by immersion of the sardine fillets in each type of gel for approximately 10 s at 45 °C and the excess coating was drained for 2 s before the fillets were stored. Additionally, sardine fillets were also coated with gels containing 2% RE as a potential antioxidant and antimicrobial agent. Four different sardine fillets treatments were obtained, one with RE into the oleogel (OR), one into the hydrogel (HR), one in the hydrogel phase of bigel (BGHR) and another into the oleogel phase of bigel (BGOR). Finally, all treatments were stored in sterile, plastic petri dishes at 4 °C for seven days. The different coating formulations and their respective composition is shown in Table 2.

### 4.3. Chemical Analysis

#### 4.3.1. Thiobarbituric Acid (TBA) Analysis

The TBA (2-thiobarbituric acid) test is a valuable chemical index of lipid oxidation, measuring malonaldehyde (MDA), a secondary lipid oxidation product. For the test, 10 g of each treatment of sardine fillets were mixed with 25 mL of deionized water, and the mixture was homogenized for 1–2 min using Ultra Turrax T18 basic (IKA Works Inc. Wilmington, NC, USA) at 14.000 rpm. Then, each sample was transferred into the distillation flask and 5 mL HCl 2N and 3–4 drops of silicone anti-foaming solution (Sigma-Aldrich, St. Louis, MO, USA) were added. Each sample was steam-distilled on a distillation unit (UDK 127, VELP Scientifica, Usmate, Italy) until 50 mL of distillate was collected. A 5 mL aliquot of the distillate was transferred into a test tube, and 5 mL of 0.02 M TBA solution was added. All samples were heated in a water bath for 35 min and then cooled with cold tap water. The absorbance at 532 nm (A532) was determined against a blank containing 5 mL of deionized water instead of the distillate, with a spectrophotometer (Shimadzu UV-1700, Europe GmbH, Duisburg, Germany). All analyses were performed in duplicate and the results were expressed as TBARs (mg MDA per kg sardine fillets). Analyses were performed when dip-coating of sardine fillets took place and on the first, second, third and fourth days of storage.

#### 4.3.2. Total Volatile Basic Nitrogen (TVB-N)

To determine total volatile basic nitrogen (TVB-N), the official EU method 95/149/EC (EC, 1995) was used. Briefly, 10 g of fish fillet were homogenized with 90 mL of 0.6 M perchloric acid (Chem-Lab NV, Zedelgem, Belgium) using an Ultra-Turrax homogenizer (IKA, Staufen, Germany). Then, the homogenate was filtered through Whatman No. 2 filter paper, and 50 mL of the filtrate was transferred into a distillation flask. The filtrate was made alkaline by the addition of 6.5 mL of 20% NaOH solution. A few drops of phenolphthalein and silicon anti-foaming agent were added to the flask to ensure sufficient alkalinization and prevent excessive foaming, respectively. Steam distillation was performed on a distillation unit (UDK 127, VELP Scientifica, Usmate, Italy) until 100 mL of distillate were collected in a flask containing 100 mL of 3 % aqueous solution of boric acid and Tashiro mixed indicator (2 g methyl-red and 1 g methylene-blue dissolved in 1000 mL 95% ethanol). TVB-N was determined by titrating the distillate with 0.01 N HCl. TVBN levels on the day the dip-coating of sardine fillets took place and on the fourth and seventh day of storage, in duplicate.

### 4.4. Microbiological Analysis

Twenty-five grams of each fish treatment was aseptically transferred into sterile stomacher bags with 225 mL of sterile Ringer solution (Ringer Solution ¼ Strength, Lab M., Limited, Lancashire, UK). The mixture was homogenized in a stomacher mixer (BagMixer 400, Interscience, St. Nom, France) for 120 s, and further appropriate dilutions were prepared for the following microorganism counts: (i) psychrotrophic counts (PTC) on Plate Count Agar (PCA, Lab M) incubated at 10 °C for seven days, (ii) *Pseudomonas* spp. on Pseudomonas Agar Base (PAB, Lab M) supplemented with cephaloridine-fucidin-cetrimide (CFC, Lab M) incubated at 25 °C for 48 h, (iii) Enterobacteriaceae on Violet Red Bile Glucose Agar (VRBGA, Lab M), incubated at 37 °C for 24 h. All microbiological counts were performed in duplicate, and the results were expressed as the log of the number of colony-forming units per g (log (CFU/g)). Microbiological analyses were conducted on the 0, first, third, fifth and seventh day of storage.

### 4.5. Statistical Analysis

All experiments were replicated twice and duplicate determinations were performed for each analysis. All the results, expressed as mean ± standard deviation, were analyzed by ANOVA, using the general linear model, at the significance level of 0.05. Differences among the samples were identified using Tukey’s multiple range test. All statistical analyses were performed using the Minitab 16 statistical software (Minitab, Inc., State College, PA, USA).

## Figures and Tables

**Table 1:** Microbiological characteristics of sardine fillets coated with or without RE during storage at 4 °C.

			Gels as Edible Coating			Gels as Edible Coating and Delivery System			
	Days	C	H	O	BG	HR	OR	BGHR	BGOR
	0	3.77 ± 0.54^a^	3.40 ± 0.08^a^	3.79 ± 0.06^a^	3.25 ± 0.15^a^	3.27 ± 0.05^a^	3.70 ± 0.14^a^	2.92 ± 0.2 5^a^	2.90 ± 0.26^a^
Psychrotrophic	1	4.74 ± 1.13^a^	4.32 ± 0.07^ab^	6.12 ± 0.03^a^	3.30 ± 0.11^b^	4.20 ± 0.18^ab^	5.87 ± 0.07^a^	3.27 ± 0.11^b^	3.22 ± 0.12^b^
Bacteria	3	7.12 ± 0.82^a^	5.00 ± 0.02^b^	7.40 ± 0.11^a^	6.74 ± 0.05^a^	5.04 ± 0.05^b^	7.42 ± 0.06^a^	6.53 ± 0.08^a^	6.12 ± 0.19^ab^
log (CFU/g)	5	8.50 ± 0.07^ab^	7.02 ± 0.07^c^	9.13 ± 0.06^a^	8.13 ± 0.11^abc^	7.03 ± 0.00^c^	8.97 ± 0.03^a^	8.03 ± 0.02^abc^	7.66 ± 0.07^bc^
	7	9.86 ± 0.22^a^	8.32 ± 0.01^d^	9.15 ± 0.06^c^	9.64 ± 0.06^ab^	8.00 ± 0.03^d^	9.00 ± 0.02^c^	9.42 ± 0.11^bc^	9.15 ± 0.16^c^
	0	3.63 ± 0.10^a^	3.22 ± 0.02^cd^	3.43 ± 0.02^abc^	3.55 ± 0.07^ab^	3.13 ± 0.02^cd^	3.34 ± 0.06^bcd^	3.29 ± 0.12^cd^	3.10 ± 0.10^cd^
*Pseudomonas* spp.	1	4.45 ± 0.47^a^	3.47 ± 0.05^b^	3.95 ± 0.06^ab^	3.95 ± 0.02^ab^	3.15 ± 0.04^b^	3.84 ± 0.07^ab^	3.82 ± 0.03^b^	3.72 ± 0.06^b^
log (CFU/g)	3	6.94 ± 0.67^a^	5.22 ± 0.12^b^	7.36 ± 0.11^a^	6.51 ± 0.11^a^	5.29 ± 0.07^b^	7.24 ± 0.01^a^	6.39 ± 0.06^ab^	6.20 ± 0.07^ab^
	5	8.28 ± 0.74^abc^	7.19 ± 0.11^d^	9.19 ± 0.09^a^	7.89 ± 0.04^bcd^	7.09 ± 0.02^d^	8.71 ± 0.15^ab^	7.43 ± 0.04^cd^	7.19 ± 0.07^d^
	7	9.83 ± 0.18^a^	9.25 ± 0.04^bc^	9.40 ± 0.11^bc^	9.74 ± 0.05^a^	9.19 ± 0.06^bc^	9.04 ± 0.08^c^	9.46 ± 0.04^b^	9.27 ± 0.05^bc^
	0	2.48 ± 0.41^a^	1.92 ± 0.11^ab^	2.46 ± 0.06^ab^	2.52 ± 0.09^a^	1.69 ± 0.12^b^	2.22 ± 0.15^ab^	2.39 ± 0.06^ab^	2.21 ± 0.08^ab^
*Enterobacteriaceae*	1	3.26 ± 0.74^a^	2.45 ± 0.03^a^	3.74 ± 0.09^a^	2.94 ± 0.03^a^	2.33 ± 0.07^a^	3.56 ± 0.08^a^	2.84 ± 0.06^a^	2.75 ± 0.08^a^
log (CFU/g)	3	5.93 ± 0.52^a^	4.40 ± 0.11^bc^	4.58 ± 0.02^bc^	5.30 ± 0.12^ab^	4.38 ± 0.01^bc^	4.30 ± 0.06^c^	5.11 ± 0.06^bc^	4.91 ± 0.05^bc^
	5	6.96 ± 0.86^ab^	6.52 ± 0.01^abc^	7.75 ± 0.04^a^	5.92 ± 0.26^bc^	6.48 ± 0.02^abc^	7.69 ± 0.02^a^	5.23 ± 0.08^c^	5.00 ± 0.04^c^
	7	8.40 ± 0.30^a^	7.59 ± 0.03^b^	7.17 ± 0.01^bc^	8.57 ± 0.06^a^	7.42 ± 0.08^b^	6.72 ± 0.01^c^	8.43 ± 0.04^a^	8.19 ± 0.08^a^

Values represent means ± standard error. ^a–d^ Different letters in the same row indicate significant differences (*p* < 0.05). C: control-uncoated fillets; H: hydrogel coating; O: oleogel coating; BG: bigel coating; HR: hydrogel coating with RE; OR: oleogel coating with RE; BHRG: coating with added RE in aqueous phase; BGOR: bigel coating with added RE in lipid phase.

**Table 2:** Composition and coding of the different gel coatings of the refrigerated sardine fillets.

Coding	Composition of Coatings	Total RE * in the Coating
C	No coating	0%
H	Hydrogel: 10% *w*/*w* gelatin in H_2_O	0%
O	Oleogel: 15% *w*/*w* monoglycerides in sunflower oil	0%
BG	20% oleogel + 80% hydrogel	0%
HR	Hydrogel: 10% *w*/*w* gelatin in H_2_O	2%
OR	Oleogel: 15% *w*/*w* monoglycerides in sunflower oil	2%
BGHR	20% oleogel + 80% hydrogel	2% (added in the aqueous phase)
BGOR	20% oleogel + 80% hydrogel	2% (added the lipid phase)

* RE = rosemary extract.

## Data Availability

The data presented in this study are available on request from the corresponding author.
